# Europium Complex-Loaded Albumin Nanoparticles as Probes for Time-Resolved Luminescent Immunoassay

**DOI:** 10.3390/bios15110761

**Published:** 2025-11-17

**Authors:** Zarina Galaeva, Maria Bochkova, Mikhail Rayev, Pavel Khramtsov

**Affiliations:** 1 Institute of Ecology and Genetics of Microorganisms, Ural Branch of RAS, Perm 614081, Russia; galaevazarina@gmail.com (Z.G.); krasnykh-m@mail.ru (M.B.); mraev@iegm.ru (M.R.); 2 Biology Faculty, Perm State University, Perm 614990, Russia

**Keywords:** lanthanide, nanoparticles, luminescence, immunoassay, albumin, immunoglobulins, reproducibility

## Abstract

We report the first analytical application of albumin nanoparticles loaded with luminescent europium complexes for immunoassay development. These nanoparticles, synthesized via a desolvation method, exhibited a uniform spherical morphology with a hydrodynamic diameter of 263 nm and strong, long-lived luminescence at 615 nm (λex = 360 nm). Surface functionalization with streptavidin enabled specific binding to biotinylated proteins. The nanoparticles were applied as labels in a sandwich time-resolved solid-phase immunoassay for human IgG detection in black 96-well plates. Unlike commercial DELFIA assays, the method eliminates the need for signal enhancement steps, as the nanoparticles intrinsically contain high concentrations of europium complexes. Optimization studies revealed that the sharp emission peaks of europium can compromise assay reproducibility; however, employing surface scanning and increasing measurement replicates per well partially mitigated this effect. Time-resolved detection reduced background by two orders of magnitude and increased signal intensity nearly tenfold in IgG-positive samples. The assay demonstrated minimal cross-reactivity with IgA and IgM (~2%) and enabled IgG detection at serum dilutions up to 1:100,000. Comparative analysis showed strong concordance with commercial immunoassays and no concentration-dependent bias. The primary limitation observed was suboptimal intra-assay reproducibility (CV > 20% in four of six tested sera).

## 1. Introduction

Luminescent bioassays have advanced considerably with the development of a wide array of nanoparticle systems, such as upconverting lanthanide-based nanoparticles, quantum dots, carbon/graphene dots, metal nanoclusters, polymer nanoparticles, among others [1,2]. Of these, polymer-based nanoparticles have attracted particular interest owing to their structural diversity and the ease with which their properties can be tuned. In particular, polymer nanoparticles incorporating luminescent moieties are emerging as highly promising platforms for bioanalytical applications [3]. From the perspective of bioassay implementation, polymer nanoparticles offer several distinct advantages: they display robust structural and colloidal stability and possess a versatile surface chemistry that enables efficient functionalization with recognition elements for selective analyte detection [4].

Among the diverse polymers utilized for nanoparticle synthesis, serum albumin—a naturally occurring biopolymer—stands out as a particularly promising candidate due to its unique physicochemical characteristics [5]. Albumin nanoparticles exhibit excellent colloidal stability, which is essential for practical applications. Moreover, albumin possesses a variety of functional groups (e.g., primary amines, carboxylates, and thiols), which facilitate chemical modification and conjugation to recognition molecules [6]. Importantly, from a translational standpoint, albumin nanoparticles represent one of the few nanoparticle systems currently approved for in vivo use and have already entered clinical practice [7]. Scalable preparation methods such as desolvation allow for the production of highly concentrated albumin nanoparticle suspensions (up to tens of milligrams per milliliter), supporting their suitability for large-scale manufacturing [8,9].

Beyond their structural merits, albumin nanoparticles also function as carriers for hydrophobic compounds due to the intrinsic binding pockets within albumin molecules. This property has been widely leveraged in theranostic applications, where albumin nanoparticles serve as vehicles for hydrophobic drugs and dyes [10]. For example, albumin nanoparticles have been used to encapsulate a range of dyes suitable for bioimaging including phthalocyanines [11], indocyanine green (ICG) [12], squaraine dyes [13], IR1061 [14], aggregation-induced emission (AIE) dyes [15,16], as well as luminescent gold nanoclusters [17]. Encapsulation within albumin nanoparticles not only enhances the colloidal stability of these dyes but also protects them from external quenchers or solvents, thereby preserving their optical properties [18]. While albumin nanoparticles have been extensively investigated in theranostic contexts [10], their potential utility in in vitro diagnostic applications remains comparatively underexplored.

Recently, our research group reported the synthesis of albumin nanoparticles encapsulating luminescent europium complexes—an innovative approach that synergistically combines the advantageous properties of both systems [19]. Europium complexes with organic ligands are well recognized for their long-lived luminescence and narrow emission bands, which are spectrally separated from the intrinsic autofluorescence of biological samples. These features significantly improve signal-to-noise ratios by minimizing background interference [20]. Owing to these properties, europium complexes are particularly well suited as labels in time-resolved luminescent immunoassays [21], and have already found use in several commercial platforms [22].

Encapsulating hydrophobic europium complexes within albumin nanoparticles provides a hydrophilic shell, thereby enhancing their aqueous compatibility and shielding the europium coordination environment from disruptive solvent interactions. Moreover, each nanoparticle can accommodate numerous europium complexes, resulting in a significant amplification of signal intensity per binding event compared to conventional labeling strategies [23,24]. Notably, unlike commercial lanthanide assays such as DELFIA, these nanoparticles do not require an enhancer solution to induce luminescence [22].

In our previous work, we thoroughly characterized the physicochemical properties of these europium-loaded albumin nanoparticles. Building on this foundation, the present study for the first time evaluates their suitability for solid-phase time-resolved luminescent immunoassay (TRLI). Specifically, we functionalized the nanoparticles with streptavidin and developed an immunoassay for human IgG detection in blood serum. During assay development, we identified potential challenges associated with lanthanide-loaded nanoparticles and proposed possible solutions. Finally, we compare the performance of our TRLI system to two commercial immunoassay platforms.

## 2. Materials and Methods

### 2.1. Materials

Bovine serum albumin was from PanEco (Moscow, Russia). 1,10-phenanthroline (further referred to as **phen**), casein, boric acid, D-biotinoyl-ε-aminocaproic acid-N-hydroxysuccinimide ester, sodium dodecyl sulfate (SDS), and beta-mercaptoethanol were obtained from (Burlington, MA, USA). Europium (III) chloride hexahydrate was from Chemcraft (Kaliningrad, Russia). Glutaraldehyde (50%), tetramethylbenzidine, HEPES, tetrabutylammonium borohydride, citric acid was from Macklin (Beijing, China). Sodium salt of EDTA, disodium phosphate, urea, TRIS, sodium carbonate, sodium chloride, and sodium azide were from Dia-M (Moscow, Russia). ELISA-1 was from Vektor-Best (Novosibirsk, Russia). ELISA-2 was from Xema (Moscow, Russia). Polyclonal antibodies against human IgG, human IgA, human IgM, and peroxidase-streptavidin conjugate were from Imtek (Moscow, Russia). Ammonium chloride and citric acid were from Helicon (Moscow, Russia). Sodium hydroxide was from Rushim (Moscow, Russia). Dimethylacetamide was from EKOS-1 (Moscow, Russia). Dimethylsulfoxide was from the Usolye-Siberian chemical-pharmaceutical plant (Usolye-Sibirskoye, Russia). Acetic acid, methanol, and hydrochloric acid were from Vekton (Moscow, Russia). Reagents for SDS-PAGE were from Bio-Rad (Hercules, CA, USA). Methyl ester of p-toluoylpyruvic acid, further referred to as **L** was kindly provided by Dr. Ekaterina Khramtsova (Faculty of Chemistry, Perm State University, Perm, Russia). Streptavidin was from ProspecBio (Rehovot, Israel). Sepharose-protein G was from Biospecifika (Novosibirsk, Russia). Tween-20 was from ITW Reagents (Monza, Italy).

Biotinylated, fluorescein-labelled oligoDNA (Bi-DNA-FAM) was purchased from Eurogen (Moscow, Russia). The sequence of the probe was as follows: 5′-biotin-TTTTTAATTAAAGCTCGCCATCAAATAGCTTT-FAM-3′. The oligoDNA (at a concentration of approximately 100 μmol/L) was stored in 10 mmol/L TRIS-HCl buffer, pH 7.4, supplemented with 0.1 mol/L Na_2_-EDTA at −25 °C. Prior to use, the probe was thawed, heated at + 90 °C for 5 min, and then allowed to cool to room temperature. The concentration was determined by measuring absorbance at 260 nm, using OligoCalc v.3.19 (http://oligocalc.eu/. Accessed 10 August 2025).

### 2.2. Instrumentation

The Multiskan Sky UV-Vis reader was from Thermo Scientific (Waltham, MA, USA). The ZetaSizer NanoZS particle analyzer was from Malvern Instruments (Malvern, UK). The VCX-130 ultrasonic processor was from Sonics & Materials (Newtown, CT, USA). The peristaltic pump P-1 was from Pharmacia (Uppsala, Sweden). The peristaltic pump BT100S was from Lead Fluid Technology (Baoding, China). Biotek Synergy H1 was from BioTek Instruments (Winooski, VT, USA), BeNano 180 Zeta Pro was from Bettersize Instruments (Dandong, China)

### 2.3. Synthesis of Eu@BSA

Bovine serum albumin (BSA) stock solutions were prepared at a concentration of 50 mg/mL in deionized water and stored at +4 °C. The concentration of BSA was determined by UV spectrophotometry at 280 nm, using an extinction coefficient such that a 1 mg/mL BSA solution yields an absorbance of 0.67 [25]. The pH of the BSA solution was adjusted to 9 with 1 mol/L NaOH.

Nanoparticle synthesis was performed in 30 mL glass vials. To initiate synthesis, 4 mL of the BSA stock solution was transferred to a vial and heated to +35 °C under constant magnetic stirring at 1000 rpm. The temperature of the solution was continuously monitored using a temperature probe.

Separately, europium(III) chloride hexahydrate, 1,10-phenanthroline (**phen**), and ligand **L** were dissolved in 95% ethanol to final concentrations of 2 mmol/L, 2 mmol/L, and 6 mmol/L, respectively. The resulting ethanolic solution (8.1 mL) was added dropwise to the pre-heated BSA solution using a peristaltic pump while maintaining the reaction conditions described above. The addition was continued until the suspension reached constant turbidity, as assessed visually.

Following synthesis, the nanoparticle suspension was heated at +70 °C for 2 h under continuous stirring, then allowed to cool to room temperature. The suspension was divided into ~1 mL aliquots for purification. Each aliquot was washed three times with deionized water by centrifugation at 20,000× *g* for 20 min. After each centrifugation step, the pellet was resuspended in deionized water using probe sonication (3 mm tip diameter, 60% amplitude, 8 W power, 15 s duration). After the final wash, all aliquots were combined and subjected to further probe sonication on ice for 20 min (6 mm tip diameter, 60% amplitude, actual power: 18–25 W). The final volume was adjusted to 15 mL with deionized water, and the nanoparticle concentration was determined by gravimetric analysis as described in Section 2.5. The resulting nanoparticle suspension was stored at +4 °C until further use.

### 2.4. Synthesis of Eu@BSA/Str

Eu@BSA nanoparticles were diluted to a final concentration of 5 mg/mL. An equal volume of 25% glutaraldehyde (pH 7.00, adjusted with 1 M NaOH) was added to the suspension. The mixture was incubated at +37 °C for 30 min on a rotator at 10 rpm with constant mixing, taking care to avoid foaming. Immediately after incubation, the absorbance at 450 nm was measured to determine the nanoparticle concentration following glutaraldehyde addition. The nanoparticles were then washed three times by centrifugation at 20,000× *g* for 20 min. After the final wash, the Eu@BSA nanoparticles were resuspended in HEPES-HCl buffer (pH 7.0), and streptavidin solution (10 mg/mL) was added to achieve a 1:10 mass ratio of streptavidin to Eu@BSA. The mixture was incubated at +37 °C for 16 h on a rotator. Excess streptavidin was removed by centrifugation at 20,000× *g* for 20 min. The resulting Eu@BSA/Str nanoparticles were washed three times with water and stored in an aqueous solution containing 1% BSA and 20% glycerol. Concentration of nanoparticles was estimated by measuring absorbance at 450 nm at different synthesis stages.

In the reproducibility experiment, a new batch of Eu@BSA nanoparticles was synthesized as described in Section 2.3. This batch was used to prepare three separate batches of streptavidin-modified nanoparticles, designated as Eu@BSA/Str-1, Eu@BSA/Str-2, and Eu@BSA/Str-3. In addition, control nanoparticles modified with BSA instead of streptavidin (Eu@BSA/BSA) were prepared to confirm the specificity of the TRLI assay.

### 2.5. Characterization of Nanoparticles

SEM and TEM images were obtained using Merlin (Carl Zeiss, Germany) and Hitachi HT7700 Excellence (Hitachi, Japan). A sample of Eu@BSA/Str without stabilizers (BSA and glycerol) was used for analysis. Nanoparticles were diluted in water to 10–1000 ng/mL before measurements.

Zeta potential was measured using BeNano 180 Zeta Pro in 10 mmol/L Sodium phosphate buffer, pH 7.0 at 10 μg/mL in auto mode. Hydrodynamic diameter was measured using Malvern ZetaSizer NanoZS (100 μg/mL in water).

Luminescence measurements were performed on aqueous suspensions of nanoparticles at concentrations of 1 and 10 μg/mL, using black 96-well plates (100 μL per well), with each condition measured in triplicate. The excitation wavelength was set to 360 nm, emission was collected at 615 nm, with a delay time of 100 μs, acquisition time of 300 μs, and a gain setting of 200 a.u.

The concentration of Eu@BSA nanoparticles was determined by gravimetric analysis. Briefly, 1 mL aliquots of Eu@BSA aqueous suspension were placed in three porcelain crucibles and heated at +95 °C to evaporate water. The crucibles were then maintained at +105 °C until a constant weight was achieved (defined as a weight difference of less than 0.2 mg between consecutive measurements). The mean concentration of Eu@BSA was calculated from these measurements.

For biotin binding experiments, Eu@BSA/Str-1 or Eu@BSA/BSA nanoparticles and Bi-DNA-FAM were added to 10 mmol/L TRIS-HCl buffer (pH 7.4) supplemented with 0.15 mol/L NaCl, 1% BSA, and 0.1% Tween-20, to a final volume of 400 μL and a nanoparticle concentration of 0.1 mg/mL. Bi-DNA-FAM was added at concentrations ranging from 0 to 400 nmol/L. Samples were incubated in a deep-well (2.2 mL well volume) 96-well plate at room temperature (+28 °C) for 60 min. After incubation, 300 μL of each sample was transferred to 1.5 mL microcentrifuge tubes and centrifuged for 30 min at 20,000× *g*. Supernatants (100 μL) were then transferred to black 96-well plates and fluorescence was measured at 485/515 nm. Calibration samples were prepared in parallel using water in place of the nanoparticle suspension.

### 2.6. Standard of Human IgG

We used pharmacy-grade affinity purified IgG fraction for intramuscular injections (95–105 mg/mL in 300 mmol/L glycine, >97% purity) as a standard.

In some experiments, for comparison, we used lyophilized human IgG (>95% purity) from Sigma Aldrich (Burlington, MA, USA). After solubilization of IgG an insoluble fraction was observed, which was removed by centrifugation at 20,000× *g* for 30 min.

Precise concentration of IgG was estimated by measuring absorbance at 280 nm using known extinction coefficient of 1.4 for 1 mg/mL solution. In addition, concentration of IgG from both manufacturers was measured using two commercial ELISAs.

### 2.7. Preparation of Human Sera Samples

Human serum samples were obtained from a laboratory biobank collection. The samples were originally collected in 2014 as part of a previous scientific project and had been stored at −25 °C until use. Prior to analysis, samples were thawed at room temperature and centrifuged at 20,000× *g* for 30 min to remove any particulate matter. Samples exhibiting visible signs of aggregation or hemolysis were excluded from further analysis. After centrifugation, serum samples were stored at +4 °C for up to four days before use in assays. A pooled serum sample was prepared by combining equal volumes of sera from five male and five female healthy volunteers (aged 18–50 years). Sodium azide was then added to the pooled sample to a final concentration of 0.01% (*w*/*v*) as a preservative.

This research was performed according to World Medical Association’s Declaration of Helsinki and Council of Europe Protocol to the Convention on Human Rights and Bio-medicine and approved by the Ethics Committee of the Institute of Ecology and Genetics of Microorganisms, Ural Branch of the Russian Academy of Sciences (IRB00010009). Written informed consent was obtained from all the participants.

### 2.8. Biotinylation of Mouse Monoclonal Antibodies

Antibodies (5.6 mg/mL, 200 μL) were dialyzed two times against 1 L of 0.1 mol/L sodium borate buffer, pH 8.8. To antibodies solution 3.45 μL of 10 mg/mL of D-biotinoyl-ε-aminocaproic acid-N-hydroxysuccinimide ester was added. After overnight incubation at +4 °C with constant stirring, 20 μL of 1 mol/L NH_4_Cl was added. After 10 min of incubation antibodies were dialyzed against a 10 mM TRIS-HCl buffer (pH 7.4) containing 10 mmol/L of NaCl and 0.01% of NaN_3_. Concentration of antibodies was measured by Bradford assay using IgG as a calibrator.

### 2.9. IgG Depletion from Human Serum

A chromatography column (internal diameter: 10 mm) was packed with 1 mL of Sepharose CL-4B covalently coupled to recombinant protein G lacking the albumin-binding domain. The resin was packed at a flow rate of 0.3 mL/min, and all subsequent elution steps were performed at 0.15 mL/min. Prior to sample application, the column was sequentially washed with phosphate-buffered saline (PBS), followed by 0.1 mol/L glycine-HCl buffer (pH 2.7), and then equilibrated with PBS.

One milliliter of pooled human serum was loaded onto the column. Protein-rich fractions (0.3 mL each) were collected based on protein content determined by the Bradford assay (5 μL sample + 250 μL Bradford reagent). Bound IgG was eluted with 0.1 mol/L glycine-HCl buffer (pH 2.7), and the eluate was immediately neutralized with 1 mol/L TRIS-HCl (pH 9.0). The IgG depletion procedure was repeated on the resulting IgG-depleted serum to ensure maximal removal of IgG. Notably, during the second depletion cycle, no protein was detected in the acidic eluate by the Bradford assay, indicating that the majority of IgG had been removed during the initial round.

The final volume of IgG-depleted serum obtained was 6 mL, corresponding to a six-fold dilution relative to the original serum. This dilution factor was accounted for during subsequent assay optimization.

IgG concentrations in both untreated and protein G-depleted sera were determined using two commercial ELISA kits as well as a custom-developed in-house ELISA. Due to the relatively high detection limits of commercial kits, IgG levels in depleted serum samples were below their quantification range. Therefore, a sensitive in-house ELISA was developed, which allowed for reliable quantification of residual IgG in depleted samples, despite some discrepancies in absolute values compared to commercial assays. Overall, IgG depletion resulted in a reduction in IgG concentration by four orders of magnitude, which was deemed satisfactory based on optimized calibration curves.

Additionally, serum samples were analyzed by SDS-PAGE (see Section 2.10), which confirmed a marked decrease in the intensity of the 150 kDa band corresponding to IgG in depleted sample compared to untreated serum (Figure S1).

### 2.10. SDS-PAGE Analysis

Sodium dodecyl sulfate polyacrylamide gel electrophoresis (SDS-PAGE) was performed using gels composed of an 8% polyacrylamide resolving layer and a 4.5% stacking layer, with a total gel thickness of 0.75 mm. Prior to loading, samples (10 μL) were mixed with 2 μL of loading buffer lacking β-mercaptoethanol. Electrophoresis was conducted at a constant voltage of 100 V per gel. Following separation, the gels were stained with Coomassie Brilliant Blue and subsequently destained using a methanol–acetic acid–water solution. The stacking gel was removed prior to imaging. Gels were scanned and the resulting images are presented without further modification.

### 2.11. In-House Human IgG ELISA

A 96-well microplate was coated with 100 μL of polyclonal rabbit antibodies against human IgG (100 ng/mL) diluted in 0.1 mol/L sodium carbonate buffer (pH 9.5) and incubated at +37 °C for 120 min on a shaker set to 350 RPM. Following the coating step, the wells were washed three times with 350 μL of PBST (phosphate-buffered saline containing 0.1% Tween-20). To block nonspecific binding sites, 250 μL of 1% casein in PBST (blocking solution) was added to each well, and the plate was incubated at +37 °C for 60 min on a shaker set to 350 RPM. After blocking, the wells were washed again with PBST. Subsequently, 100 μL of human IgG diluted in the blocking solution was added to each well, followed by incubation at +37 °C for 60 min on a shaker. The wells were then washed, and 100 μL of biotinylated mouse monoclonal antibodies against human IgG (100 ng/mL) diluted in the blocking solution was added. The plate was incubated at +37 °C for another 60 min on a shaker and subsequently washed. Next, 100 μL of horseradish peroxidase (HRP)–streptavidin conjugate diluted in the blocking solution (1:50,000) was added to the wells. The plate was incubated at +37 °C for 60 min on a shaker and washed thoroughly with PBST. For detection, 100 μL of TMB substrate solution was added to each well. The TMB substrate solution was prepared by mixing 8 mL of 0.205 mol/L potassium citrate buffer (pH 4.0) containing 3.075 mmol/L H_2_O_2_ with 200 μL of a stock solution containing 41 mmol/L TMB and 8.2 mmol/L tetrabutylammonium borohydride in dimethylacetamide [26]. The reaction was allowed to proceed for 20 min before being stopped by adding 2 mol/L H_2_SO_4_. The absorbance at 450 nm was measured using a plate spectrophotometer.

### 2.12. Time-Resolved Luminescent Immunoassay for Human IgG

The optimized protocol for the time-resolved luminescent immunoassay of human immunoglobulin G (IgG) is described below. Unless otherwise specified in the Results section, human IgG standards were diluted in blocking solution without the addition of IgG-depleted human serum.

A 96-well microplate was coated with 100 μL per well of polyclonal rabbit anti-human IgG antibodies (1 μg/mL) prepared in 0.1 mol/L sodium carbonate buffer (pH 9.5), and incubated overnight at +4 °C. Following the coating step, wells were washed three times with 350 μL of TBST (10 mmol/L TRIS-HCl, pH 7.4, containing 0.1% Tween-20). To block nonspecific binding sites, 250 μL of 1% casein in TBST (blocking solution) was added to each well, and the plate was incubated at +37 °C for 60 min on a shaker at 350 rpm. After blocking, wells were washed again with TBST.

Subsequently, 100 μL of sample diluted in blocking solution containing 0.001% IgG-depleted pooled human serum was added to each well, followed by incubation at +37 °C for 60 min with shaking. After washing, 100 μL of biotinylated mouse monoclonal anti-human IgG antibodies (500 ng/mL in blocking solution) was added to each well. The plate was incubated at +37 °C for an additional 60 min on a shaker and washed as described above.

Next, 100 μL of Eu@BSA/Str conjugate (50 μg/mL in blocking solution) was dispensed into each well. The plate was incubated at +20 °C for 60 min with shaking and then thoroughly washed with PBST.

For detection, 100 μL of 5 mol/L aqueous urea solution was added to each well. Time-resolved luminescence intensity was measured in a bottom scanning mode using an excitation wavelength of 360 nm and emission at 615 nm, with a delay time of 100 μs and an acquisition time of 300 μs (gain: 200; five points per well; 50 measurements per point).

### 2.13. Data Analysis

Analyte concentrations in the ELISA assays were determined using point-to-point linear regression equations, in accordance with the manufacturers’ instructions. Limits of detection (LODs) were back-calculated from standard curves using the luminescence intensity corresponding to the mean blank signal plus three standard deviations. The same approach was employed for the luminescent immunoassay in the comparative studies. In certain experiments, a five-parameter logistic (5PL) curve fitting was performed using Origin 2020b software (OriginLab Corporation, Northampton, MA, USA) to illustrate issues related to curve fitting arising from poor intra-plate reproducibility.

## 3. Results and Discussion

### 3.1. Synthesis and Characterization of Nanoparticles

**Figure 1:**
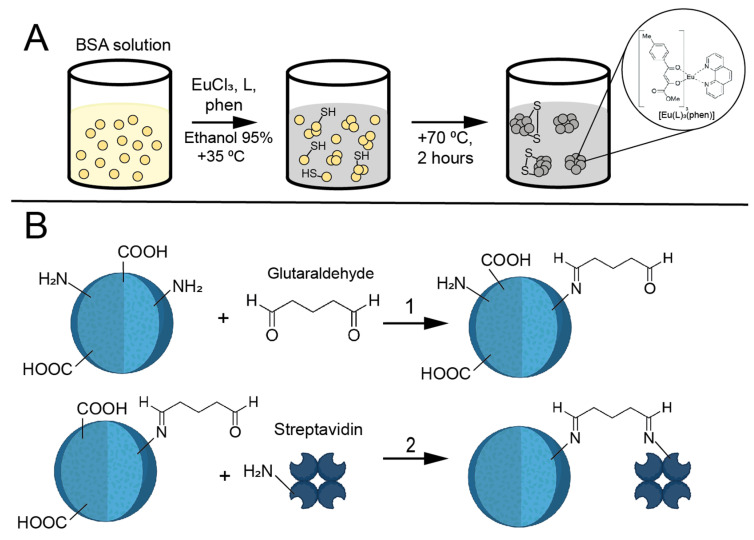
(**A**) Schematic representation of the synthesis of Eu@BSA nanoparticles. (**B**) Functionalization of Eu@BSA nanoparticles with streptavidin.

In the initial phase of our study, we synthesized luminescent BSA nanoparticles encapsulating europium complexes via the desolvation method (Figure 1A) [19]. Briefly, organic ligands **L** and **phen**, together with EuCl_3_, were dissolved in 95% ethanol, and this solution was added dropwise to an aqueous BSA solution under alkaline conditions. The addition of ethanol reduced the solubility of BSA, resulting in nanoparticle formation. Simultaneously, the ligands and europium ions reacted in the alkaline medium to form luminescent **[Eu(L)_3_(phen)]** complexes, which became entrapped within the developing nanoparticles. The structural integrity of the nanoparticles was further enhanced by heating at +70 °C for 2 h, promoting intermolecular disulfide bond formation and cross-linking of the BSA matrix. The resulting Eu@BSA nanoparticles were then treated with an excess of glutaraldehyde to modify surface amino groups with aldehyde functionalities, thereby enabling subsequent covalent conjugation of streptavidin (Eu@BSA/Str) (Figure 1B). Glutaraldehyde typically reacts with two primary amine groups located within approximately 0.75 nm of each other; however, its oligomeric forms can facilitate cross-linking over greater distances [27]. This bifunctional aldehyde is widely employed to stabilize albumin nanoparticles prepared via the desolvation method. Glutaraldehyde reacts with primary amines on proteins, and when used in excess, some molecules may react with only a single amine group, leaving the second aldehyde group available for subsequent conjugation with other amine-containing molecules.

**Figure 2:**
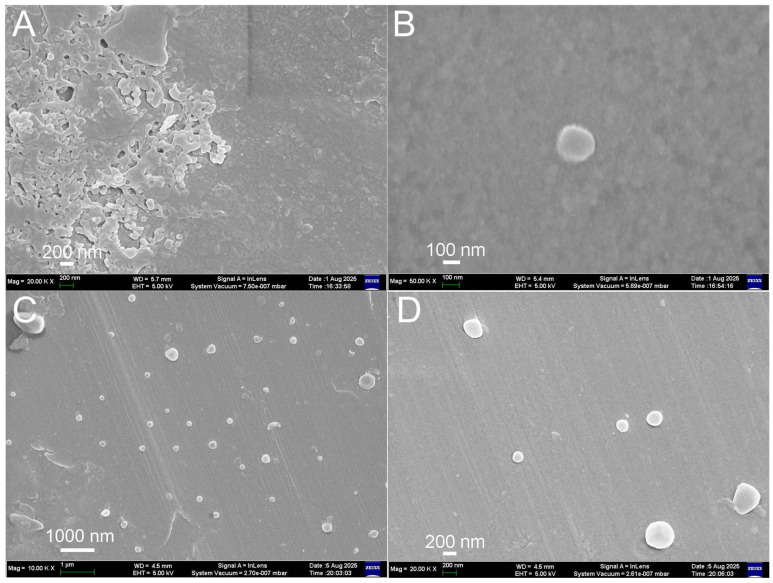
SEM images of (**A**,**B**) Eu@BSA nanoparticles and (**C**,**D**) Eu@BSA/Str nanoparticles.

Key properties of Eu@BSA, Eu@BSA/Str, and the glutaraldehyde-treated intermediate are summarized in Table 1. The synthesized nanoparticles exhibited pronounced red luminescence upon ultraviolet illumination. Representative excitation and emission spectra are presented in Figure S2. The mean hydrodynamic diameter of Eu@BSA/Str was 263 nm, approximately 20 nm larger than that of Eu@BSA. This increase can be attributed to the cross-linking process and the attachment of streptavidin, which has a diameter of about 5 nm [28]. The resulting Eu@BSA/Str nanoparticles exhibited a low polydispersity index, indicating high uniformity. After two months of storage, the size of Eu@BSA/Str nanoparticles remained unchanged (Dh = 256 ± 9 nm, PdI = 0.044 ± 0.023), indicating excellent colloidal stability. Scanning electron microscopy (SEM) analysis confirmed that both Eu@BSA and Eu@BSA/Str nanoparticles possessed a spherical morphology (Figure 2). Transmission electron microscopy (TEM) images of Eu@BSA (Figure S3A), however, revealed irregular snowflake-like structures, consistent with those observed for dried pure BSA solutions on aluminum substrates [29]. In contrast, such snowflake-like structures were absent in TEM images of Eu@BSA/Str (Figure S3B), although discrete nanoparticles were also not clearly visible. The limited resolution in TEM is likely due to aggregation during the drying process and the inherently low electron density of these polymeric nanostructures, which hampers the visualization of individual nanoparticles. More detailed characterization of Eu@BSA, including confirmation of complex encapsulation and physicochemical properties, has been reported in a recent paper [19].

The functionalization process was monitored by measuring both the zeta potential and the concentration-normalized luminescence intensity of the nanoparticles. Initially, the nanoparticles exhibited a negative zeta potential, consistent with the net negative charge of BSA molecules at neutral pH (isoelectric point ~4.7–5) [30]. Following glutaraldehyde treatment, the zeta potential decreased from −19.5 mV to −26.2 mV, which can be attributed to the reaction of lysine side chains with glutaraldehyde. Subsequent conjugation with streptavidin did not result in any significant change in zeta potential. In parallel, the luminescence intensity of the nanoparticles decreased by approximately 3.5-fold over the course of functionalization. As Eu@BSA nanoparticles are known to retain their luminescence over long-term storage [19], we attribute this decrease primarily to the multiple sonication steps employed during functionalization, which likely facilitated the diffusion of encapsulated complexes into the surrounding solution.

Successful conjugation of streptavidin was further confirmed by assessing the binding of biotinylated oligoDNA to Eu@BSA/Str and to control nanoparticles, Eu@BSA/BSA. As shown in Figure S4, the amount of oligoDNA bound increased with the initial oligoDNA-to-nanoparticle ratio. Across all tested concentrations, binding to streptavidin-modified nanoparticles was at least an order of magnitude higher than to controls. At the highest oligoDNA concentration, Eu@BSA/Str nanoparticles bound 1024.4 pmol/mg, compared to only 90.2 pmol/mg for Eu@BSA/BSA. Furthermore, development and optimization of a time-resolved immunoassay confirmed its specificity by comparing the performance of Eu@BSA/Str and Eu@BSA/BSA nanoparticles (see Section 3.3). These experiments also demonstrated negligible binding of biotinylated targets to nanoparticles lacking streptavidin.

### 3.2. Development of TRLI for Human IgG

#### 3.2.1. Principle of the Assay

**Figure 3:**
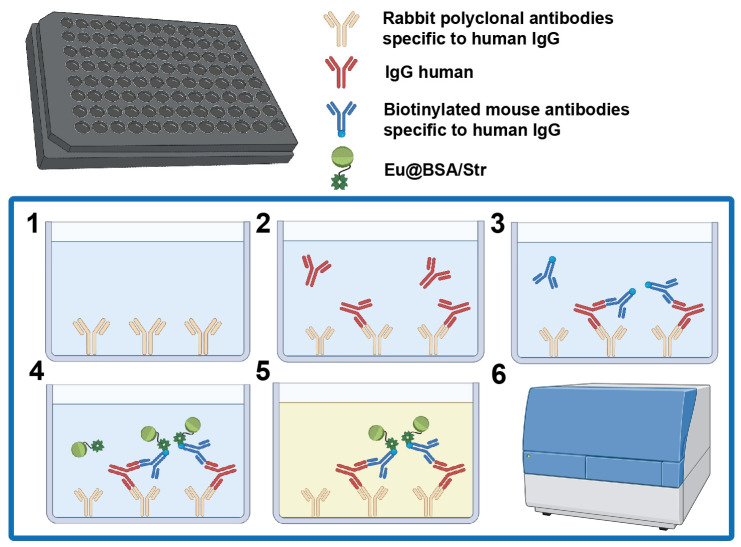
Workflow of the TRLI: (**1**) immobilization of capture antibodies; (**2**) addition of sample containing human IgG; (**3**) addition of biotinylated detection antibodies; (**4**) addition of Eu@BSA/Str nanoparticles; (**5**) addition of urea solution; (**6**) measurement of luminescence intensity at 360/615 nm with a 100 μs delay. Washing steps between assay steps are omitted for clarity.

TRLI was performed in black 96-well plates using a sandwich format, employing rabbit polyclonal antibodies as capture antibodies and either biotinylated mouse monoclonal or rabbit polyclonal antibodies as detection antibodies. Streptavidin-functionalized nanoparticles were used to bind the biotinylated secondary antibodies via the high-affinity biotin-streptavidin interaction (Figure 3, steps 1–6). Unlike the commercial europium-based DELFIA assay with its enhancement step prior to measurement [31], our assay does not require such an enhancement procedure. In our system, europium ions are already coordinated to ligands within the nanoparticles, providing intrinsic luminescent enhancement.

Detection was performed by measuring luminescence at 615 nm following excitation at 360 nm. A delay time of 100 μs was applied prior to signal acquisition, with an integration time of 300 μs. The choice of signal acquisition time was based on luminesce decay curves of Eu@BSA, which were obtained in our previous study [19] to both minimize autofluorescence and obtain high signal intensity. It is important to note that a low-molarity TRIS-HCl buffer was used as the reaction medium due to its minimal chelating properties. As previously demonstrated, phosphate and other buffers capable of binding trivalent metal cations significantly reduce nanoparticle luminescence [19]. All measurements were conducted at room temperature, as increasing temperature has been shown to decrease the luminescence intensity of Eu@BSA/Str.

#### 3.2.2. Challenges of TRLI

**Figure 4:**
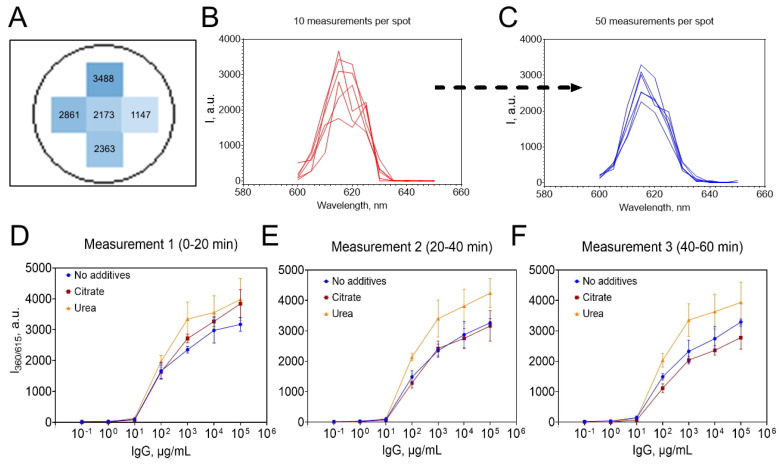
(**A**) Representative luminescence intensity values obtained in bottom scanning mode; CV for five measurements in distinct regions of a single well was 36%. Emission spectra recorded in six separate wells at (**B**) 10 and (**C**) 50 measurements per spot. Effect of different eluents on luminescence intensity measured at (**D**) 0–20 min, (**E**) 20–40 min, and (**F**) 40–60 min post-eluent addition; one plate measurement required 20 min. n = 3; mean ± SD.

A major challenge encountered during the development of the TRLI assay was the poor reproducibility of luminescence intensity across technical replicates (i.e., wells containing the same sample). We hypothesized that this variability could be attributed to the random distribution of europium nanoparticles on the surface of the microplate wells. At a constant analyte concentration, although the total number of nanoparticles per well remained unchanged, the excitation laser likely encountered varying numbers of nanoparticles depending on their spatial arrangement. This resulted in substantial fluctuations in the measured signal between replicates. Indeed, non-uniform signal distribution within individual wells was confirmed by performing luminescence measurements in well bottom scanning mode (Figure 4A).

Another source of poor reproducibility was identified as the intrinsically narrow emission band of europium. Unlike conventional fluorescent dyes, europium exhibits a sharp emission peak with a steep intensity gradient between adjacent wavelengths. Analysis of the emission spectra revealed shifts (up to 10 nm) in the peak emission wavelength (Figure 4B), which we attribute to subtle differences in the microenvironment surrounding nanoparticles immobilized on the well surface. Due to the narrowness of the europium emission peak, even these slight spectral shifts led to significant changes in the recorded luminescence intensity.

To improve the reproducibility of luminescence measurements, several modifications were implemented. First, measurements were performed in bottom scanning mode. Specifically, for each well, luminescence was measured at five distinct positions, and the mean value was used for analysis. Increasing the number of measurement spots per well did not further enhance reproducibility but substantially increased plate processing time (Figure S5). Secondly, the number of repeated measurements at each spot was increased from 10 to 50, following a methodology similar to that described by [32]. This adjustment resulted in more homogeneous and stable emission spectra, with the peak wavelength consistently observed at 615 nm and no evidence of red shift (Figure 4C). Third, we investigated the addition of various solutions to the wells prior to measurement, with the aim of dissociating immune complexes and thereby improving both signal intensity and reproducibility (Figure S6). The rationale was that elution of nanoparticles from the well surface, via dissociation of immune complexes into solution, would yield a more homogeneous distribution and increase the number of nanoparticles available for excitation. Among the tested conditions, 5 mol/L urea increased signal intensity by approximately 30% compared to empty wells or deionized water (Figure 4D–F); however, this did not improve reproducibility. Other commonly used dissociation agents—including acids, bases, and detergents—either had no beneficial effect or reduced luminescence due to disruption of the coordination bonds within the complexes. The effect of sodium citrate (pH 3) is shown in Figure 4D–F as an example. Sodium citrate is widely used for dissociating antigen–antibody complexes in affinity chromatography. When added to the wells, it increased luminescence intensity (Figure 4D). However, prolonged incubation led to a decline in signal (Figure 4E, F), likely due to competitive binding of citrate ions to Eu^3+^.

#### 3.2.3. Optimization of TRLI

The primary objective of the optimization process was to achieve an optimal balance between a high signal-to-background ratio, steep and broad calibration curve slopes, enhanced assay selectivity, and minimal reagent consumption.

**Figure 5:**
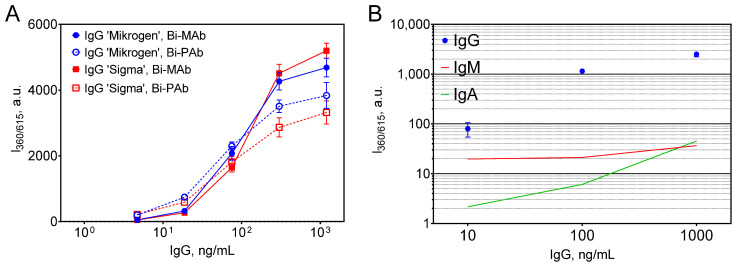
(**A**) Dose–response curves generated using human IgG from different suppliers with either biotinylated monoclonal (Bi-MAb) or polyclonal (Bi-PAb) anti-human antibodies. (**B**) Luminescence intensities at various IgG concentrations (blue circles); red and green lines indicate responses for IgM and IgA at physiological serum concentration ratios relative to IgG, e.g., at 10 ng/mL IgG, corresponding IgA concentration is 2.34 ng/mL, reflecting a 4.27-fold lower level. n = 3; mean ± SD.

Initially, the concentrations of capture and detection antibodies, as well as nanoparticles, were systematically optimized. For detection, two types of biotinylated antibodies specific to human IgG were evaluated: mouse monoclonal antibodies and rabbit polyclonal antibodies (the latter identical to the coating antibodies). Screening of assay conditions (Figure S7) indicated that the best compromise between analytical performance and reagent economy was attained using 1 μg/mL of polyclonal capture antibody, 500 ng/mL of biotinylated monoclonal detection antibody, and 50 μg/mL of Eu@BSA/Str nanoparticles (Figure S7E). Notably, substitution of the biotinylated monoclonal antibody with its polyclonal counterpart resulted in diminished signal intensity at higher analyte concentrations (>75 ng/mL) and increased signal at lower concentrations (Figure 5A). Overall, the use of biotinylated monoclonal antibodies resulted in steeper dose–response curves and lower detection limits: 1.27 ng/mL for IgG ‘Mikrogen’ and 2.48 ng/mL for IgG ‘Sigma’ with monoclonal antibodies, compared to 1.51 ng/mL and 8.18 ng/mL, respectively, with polyclonal antibodies.

Another critical factor was the selection of an appropriate human IgG calibrator. Two sources of human IgG were evaluated: (1) highly purified (>97%) ready-to-use intravenous solution (“Mikrogen”) and (2) lyophilized IgG (>95%) intended for research applications (“Sigma”). Upon dilution of the latter preparation, aggregates were observed but could be removed by centrifugation. The concentration of both preparations was determined by spectrophotometry and two commercial ELISA kits (Table S1). Interestingly, the ELISA kits from different manufacturers produced discrepant results for both IgG preparations. Given that spectrophotometry provides a direct measurement of protein concentration, its values were used to construct calibration curves. Minor differences in calibration curves were observed between the two IgG sources (Figure 5A). Ultimately, the intravenous IgG preparation was selected as the calibrator due to its lack of persistent aggregation, which could otherwise result in polymeric forms that are not removable by centrifugation and may affect assay accuracy due to altered diffusion rates, molecular size, and epitope availability compared to monomeric IgG. Furthermore, we highlight that one commercial ELISA kit (ELISA-1) consistently underestimated IgG concentration. Due to these discrepancies, subsequent experiments included a comparative analysis of luminescence immunoassay results with both ELISA kits.

Due to the structural homology among immunoglobulin classes, some degree of cross-reactivity is anticipated in immunoassays. To quantitatively evaluate the potential interference of IgA and IgM in the measurement of IgG, individual dose–response curves were established for each immunoglobulin (IgG, IgA, and IgM; Figure S8). The impact of IgA and IgM on the luminescence signal was then assessed by simulating their physiological ratios relative to IgG, based on published reference concentrations in human serum [33]. Specifically, the mean serum concentrations are reported as 11.18 g/L for IgG, 2.62 g/L for IgA (4.27-fold lower than IgG), and 1.47 g/L for IgM (7.61-fold lower than IgG). These ratios were used to calculate the corresponding concentrations of IgA and IgM at each tested IgG concentration. For instance, at a serum dilution yielding 10 ng/mL IgG, the equivalent concentrations of IgA and IgM would be 2.34 ng/mL and 1.31 ng/mL, respectively.

Using the generated dose–response data, we estimated the proportion of luminescence signal attributable to IgA and IgM at each IgG concentration. When biotinylated monoclonal anti-IgG antibodies were employed, interference from IgA did not exceed 2.7%, and from IgM remained below 2% at both 100 ng/mL and 1000 ng/mL IgG (Figure 5B). However, at the lowest tested concentration (10 ng/mL IgG), signal contribution from IgM increased to nearly 25%. In comparison, the use of polyclonal antibodies resulted in slightly higher cross-reactivity with both IgA and IgM (Tables S2 and S3).

These findings indicate that monoclonal antibodies offer superior selectivity for IgG detection under the tested conditions. Combined with their ability to generate steeper calibration curves, biotinylated monoclonal antibodies were thus selected for subsequent optimization of the TRLI assay protocol.

Although interference from IgM and IgA was found to be negligible, additional measures were implemented to further enhance the specificity of the assay. In previous experiments, IgG standards were diluted in blocking buffer (1% casein and 0.1% Tween-20 in neutral TRIS-HCl), whereas serum samples, by necessity, contain a complex mixture of endogenous proteins. This discrepancy in matrix composition may introduce matrix effects, even though the high dilution factors employed in the assay substantially reduce such risks. To minimize potential matrix-related artifacts and to address the minor cross-reactivity observed with IgM and IgA, we prepared IgG-depleted pooled human serum using affinity purification on a Sepharose-protein G column (see Experimental section for details). As this procedure selectively removes IgG while retaining other immunoglobulins, the resulting matrix was expected to more accurately reflect the composition of actual samples and thus minimize differences in luminescence signal due to IgM and IgA cross-reactivity.

**Figure 6:**
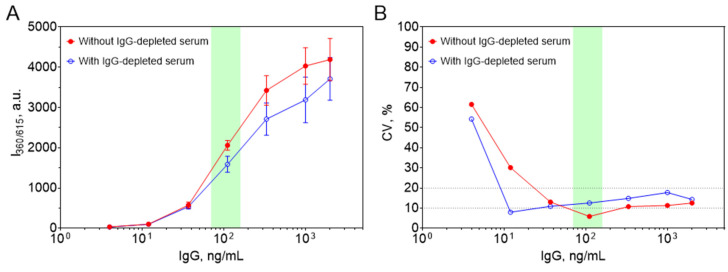
Comparison of standard curves (**A**) and coefficient of variation profiles (**B**) obtained using blocking buffer alone or buffer supplemented with IgG-depleted human serum diluted 1:100,000. The green shaded area denotes the physiological range of IgG in diluted normal human serum. n = 6; mean ± SD.

We subsequently optimized the dilution factor for serum samples. Ideally, samples should be diluted to yield an IgG concentration near 100 ng/mL, corresponding to the steepest segment of the standard curve, where both signal resolution and reproducibility are maximal and interference from non-IgG immunoglobulins is minimal. Based on the calibration curve, an optimal dilution factor of 1:100,000 was determined for serum samples. Given that typical IgG concentrations in human serum range from 7 to 16 mg/mL [33], this dilution yields final concentrations between 70 and 160 ng/mL—well within the most sensitive region of the assay (Figure 6A).

Importantly, the use of IgG-depleted serum as a diluent for standards resulted in a decrease in absolute luminescence intensity but did not affect the shape of the calibration curve, limit of detection (6.72 ng/mL for non-depleted serum and 4.83 ng/mL for IgG-depleted serum), or assay reproducibility (Figure 6A,B).

A principal advantage of time-resolved immunoassays employing lanthanide chelate labels is the ability to measure luminescence intensity several microseconds after excitation, thereby effectively eliminating short-lived autofluorescence from biological matrices. To demonstrate the impact of time-resolved detection, we compared the luminescence intensities of IgG standards measured with and without a 100 μs delay, which was routinely applied in all subsequent experiments. Implementation of the time-resolved mode resulted in an almost two orders of magnitude reduction in background signal from blank samples (Figure S9). Additionally, the signal intensity for non-zero standards increased substantially, primarily due to the integration of the europium emission over a 300 μs collection window. As a result, the use of time-resolved detection significantly improved both the slope of the standard curve and the signal-to-noise ratio, thereby enhancing assay sensitivity and analytical performance.

Using the optimized immunoassay procedure, we evaluated the batch-to-batch reproducibility of the conjugates. From a new batch of Eu@BSA, three separate conjugates were prepared under identical conditions: Eu@BSA/Str-1, Eu@BSA/Str-2, and Eu@BSA/Str-3. Their hydrodynamic diameters were 214, 217, and 224 nm, respectively (CV = 2.4%), indicating good reproducibility of the conjugation process (Figure S10A). These conjugates were also compared in TRLI (Figure S10B). Notably, all three conjugates exhibited significantly higher absolute luminescence intensities, which can be attributed to the increased luminescence of the newly prepared parent Eu@BSA (Figure S11). Control Eu@BSA/BSA nanoparticles prepared from the same parent batch generated negligible signal in TRLI (inset of Figure S10B), confirming the importance of the biotin-streptavidin interaction in the immunoassay. The limits of IgG detection in TRLI were 0.39, 0.57, and 0.28 ng/mL for Eu@BSA/Str-1, Eu@BSA/Str-2, and Eu@BSA/Str-3, respectively. We further compared luminescence intensity values generated by the three conjugates using two-way ANOVA. This analysis revealed statistically significant differences (*p* < 0.05) for some IgG concentrations, indicating that standard curves generated by these conjugates are not identical. Given that the diameters and luminescence intensities (Figure S11) of these nanoparticles were nearly identical, we suggest that variability in the conjugation procedure was responsible for these differences.

### 3.3. Comparison with Commercial ELISAs

To assess the performance of the TRLI relative to established methods, we analyzed 24 serum samples from healthy volunteers using two commercial ELISA kits (ELISA-1 and ELISA-2) as well as the TRLI method. The raw IgG concentration data are presented in Table S4.

**Figure 7:**
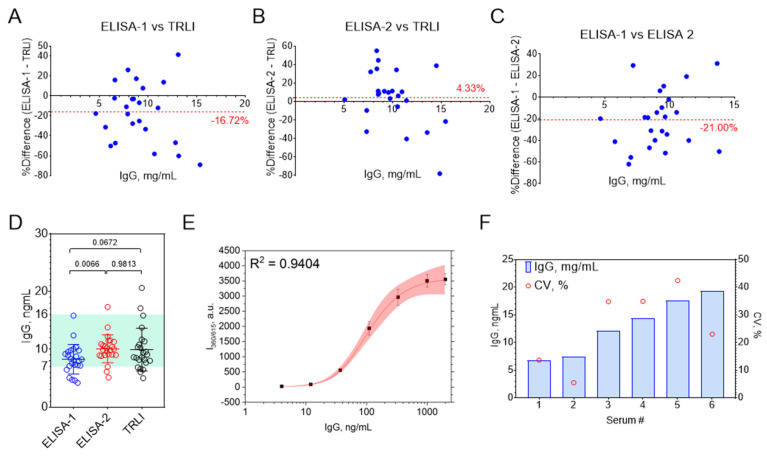
(**A**–**C**) Bland–Altman plots comparing ELISA and TRLI results; red lines and values indicate mean differences between methods. (**D**) Comparison of mean IgG concentrations measured by three methods using repeated-measures one-way ANOVA with Tukey’s post hoc correction (n = 24); lines represent means ± SD. The green shaded region indicates the normal range for human serum IgG [33]. (**E**) Five-parameter logistic fit of a representative TRLI standard curve; red area indicates the 95% confidence interval, n = 6; mean ± SD. (**F**) Coefficients of variation for IgG concentrations in six serum samples measured by TRLI, n = 6.

Pairwise method comparison using Bland–Altman analysis (Figure 7A–C) revealed good overall agreement between TRLI and ELISA-2, while ELISA-1 again demonstrated a systematic negative bias. Importantly, Bland–Altman plots indicated no systematic relationship between IgG concentration and the magnitude of differences observed between TRLI and ELISA.

Statistical comparison of mean IgG concentrations obtained by each method was performed using one-way ANOVA. Notably, a statistically significant difference (*p* < 0.05) was observed only between the two commercial ELISAs, suggesting that the differences between these assays are unlikely to be due to random variation (Figure 7D).

ELISA-1 consistently yielded lower IgG concentrations compared to both ELISA-2 and TRLI, which is consistent with our earlier findings from the analysis of IgG standards, where ELISA-1 systematically underestimated IgG concentrations. Thus, we attribute the observed discrepancies primarily to assay-specific characteristics inherent to ELISA-1.

Overall, agreement between TRLI and both commercial ELISAs was greater than that observed between the two ELISA kits themselves. However, it should be noted that absolute differences in IgG concentrations for individual samples varied substantially across the three methods, reaching up to 60% in some cases. This highlights the importance of method selection and standardization in quantitative immunoassays.

To evaluate the intra-day reproducibility of the IgG quantification, we selected six serum samples representing both high and low IgG concentrations. Each sample was analyzed in eight replicates within a single day. The results are summarized in Figure 7F. Notably, only one sample (serum #2) exhibited a coefficient of variation (CV) below 10%, which is generally considered acceptable for solid-phase immunoassays. For the remaining samples, CV values exceeded 10%, with most surpassing 20%, indicating suboptimal repeatability.

Interestingly, reproducibility was superior at lower IgG concentrations, consistent with the repeatability observed for IgG standards. This trend corresponds with improved curve fitting in the lower concentration range of the standard curve (Figure 7E). These findings are in agreement with previous reports on commercial DELFIA assays, where poor reproducibility and outlier wells with aberrant signals have also been documented [34,35]. In those studies, statistical methods such as the Dixon test were employed to exclude outliers; however, such approaches are not routinely feasible in clinical practice, and therefore all replicate results were included in our analysis without exclusion.

Similarly, elevated intra-assay CVs (>10%) have been reported for DELFIA in other studies [36], although some groups have achieved considerably better reproducibility [37,38].

In summary, while the immunoassay demonstrated good overall concordance with commercial assays, improvements in assay repeatability are warranted to enhance its suitability for routine clinical or research applications.

## Figures and Tables

**Table 1:** Physicochemical characteristics of Eu@BSA and Eu@BSA/Str nanoparticles (n = 3; data presented as mean ± SD).

Sample	Dh, nm	PdI	Zeta potential, mV	Luminescence Intensity (360/615, 1 μg/mL), a.u.
Eu@BSA	244 ± 16	0.139 ± 0.09	−19.5 ± 0.4	14,200 ± 2193
Glutaraldehyde-treated Eu@BSA	253 ± 23	0.102 ± 0.08	−26.2 ± 0.7	6484 ± 1446
Eu@BSA/Str	263 ± 5	0.051 ± 0.03	−26.1 ± 1.1	3277 ± 247

## Data Availability

Data is contained within the article or Supplementary Materials.

## Supplementary Materials

The following supporting information can be downloaded at: https://www.mdpi.com/article/10.3390/bios15110761/s1, Figure S1: SDS-PAGE analysis of IgG-depleted and native pooled human serum; Figure S2: Excitation (A) and emission (B) spectra of Eu@BSA/Str nanoparticles; Figure S3: TEM images of (A) Eu@BSA nanoparticles and (B) Eu@BSA/Str nanoparticles; Figure S4: Adsorption of Bi-DNA-FAM on Eu@BSA/Str and Eu@BSA/BSA; Figure S5: Coefficient of variation in luminescence intensity under different measurement conditions; Figure S6: Dependence of luminescence signal coefficient of variation on analyte concentration; Figure S7: Optimization of capture antibody, detection antibody, and Eu@BSA/Str nanoparticle concentrations for TRLI performance; Figure S8: Dose–response curves for IgG, IgM, and IgA in TRLI; Figure S9: Comparison of IgG standard curves acquired by measuring luminescence signal with and without a time delay; Figure S10: Reproducibility of Eu@BSA/Str properties; Figure S11: Reproducibility of Eu@BSA/Str luminescence intensity; Table S1: Concentrations of IgG standards as determined by different analytical techniques; Table S2: Effect of IgM and IgA at concentrations typical of human serum on luminescence intensity in TRLI with biotinylated monoclonal antibodies.; Table S3: Effect of IgM and IgA at concentrations typical of human serum on luminescence intensity in TRLI with biotinylated polyclonal antibodies; Table S4: Comparison of human IgG concentrations (mg/mL) in serum samples measured by ELISA and by the luminescent immunoassay.
