# Supplementary material for: Europium Complex-Loaded Albumin Nanoparticles as Probes for Time-Resolved Luminescent Immunoassay
Source: Biosensors (Basel). 2025 Nov 17;15(11):761. doi: 10.3390/bios15110761 (PMC12649969; doi:10.3390/bios15110761)
Supplement: Supplementary file 1 [file biosensors-15-00761-s001.zip › biosensors-3903271-supplementary.pdf]

biosensors

MDPI

Article

Europium Complex-Loaded Albumin Nanoparticles as Probes for Time-Resolved Luminescent Immunoassay

Zarina Galaeva [1,2], Maria Bochkova [1,2], Mikhail Rayev [1,2] and Pavel Khramtsov [1,2,*]

1 Institute of Ecology and Genetics of Microorganisms, Ural Branch of RAS, Perm, 614081, Russia; galaevazarina@gmail.com(Z.G.); krasnykh-m@mail.ru(M.B.); mraev@iegm.ru(M.R.)
2 Biology faculty, Perm State University, Perm, 614990, Russia;
* Correspondence: khramtsovpavel@yandex.ru; khramtsov.p@iegm.ru; Tel.: +7 342 280 77 94

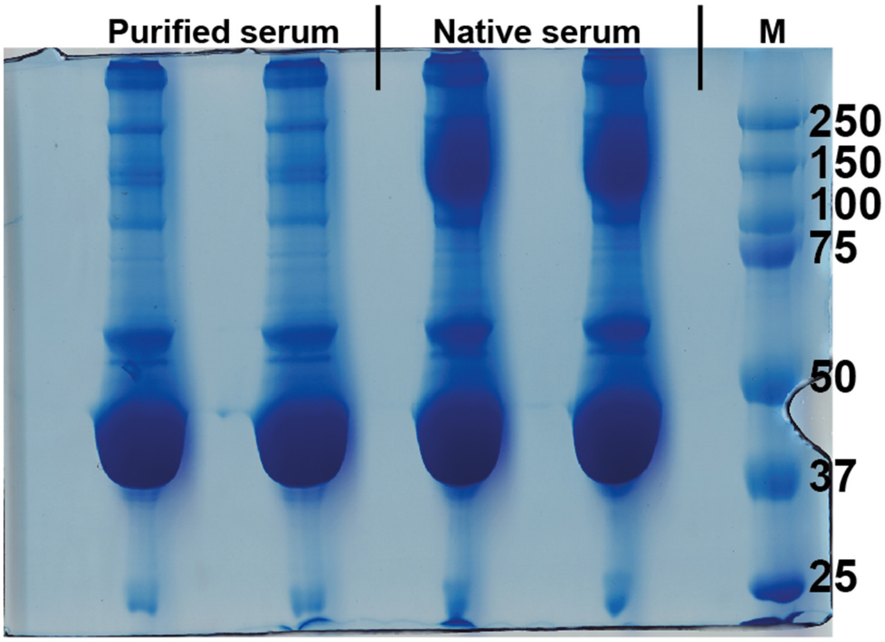

Figure S1. SDS-PAGE analysis of IgG-depleted and native pooled human serum; two replicates per sample, separated on 8% polyacrylamide gels. Lane 'M' contains molecular weight markers (kDa). The IgG band is observed at 150 kDa.

Received: 15 September 2025
Revised: 05 November 2025
Accepted: 07 November 2025
Published: date

Citation: Galaeva, Z.; Bochkova, M.; Rayev, M.; Khramtsov, P. Europium Complex-Loaded Albumin Nanoparticles as Probes for Time-Resolved Luminescent Immunoassay. To be added by editorial staff during production.

Copyright: © 2025 by the authors. Submitted for possible open access publication under the terms and conditions of the Creative Commons Attribution (CC BY) license (https://creativecommons.org/licenses/by/4.0/).

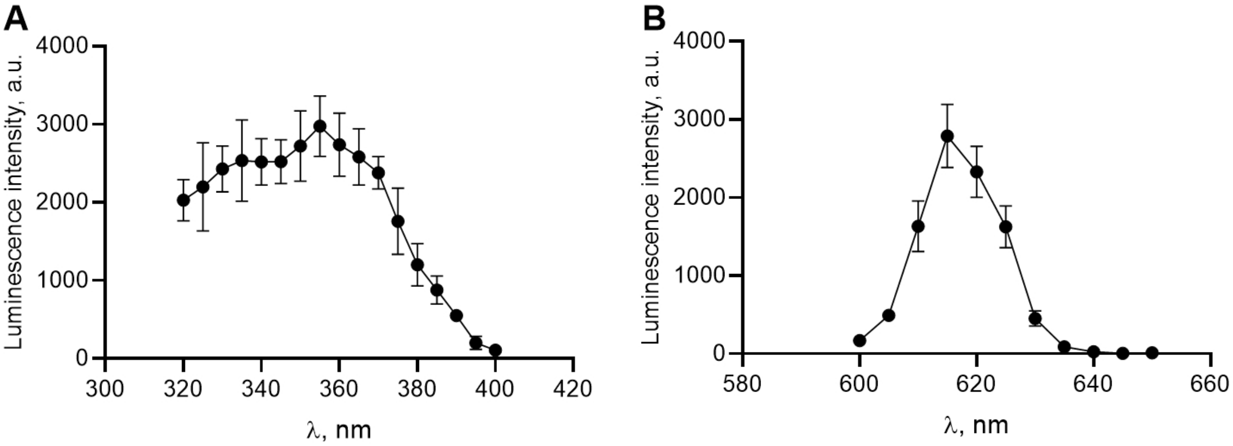

Figure S2. Excitation (**A**) and emission (**B**) spectra of Eu@BSA/Str nanoparticles. Excitation spectra were recorded from 320 to 400 nm with emission monitored at 615 nm; emission spectra were collected from 600 to 650 nm with excitation at 360 nm. Instrument gain was set to 200; each data point represents the mean of 50 measurements. Measurements were performed in TRLI well after analysis completion using the IgG calibration standard (2000 ng/mL; n = 6).

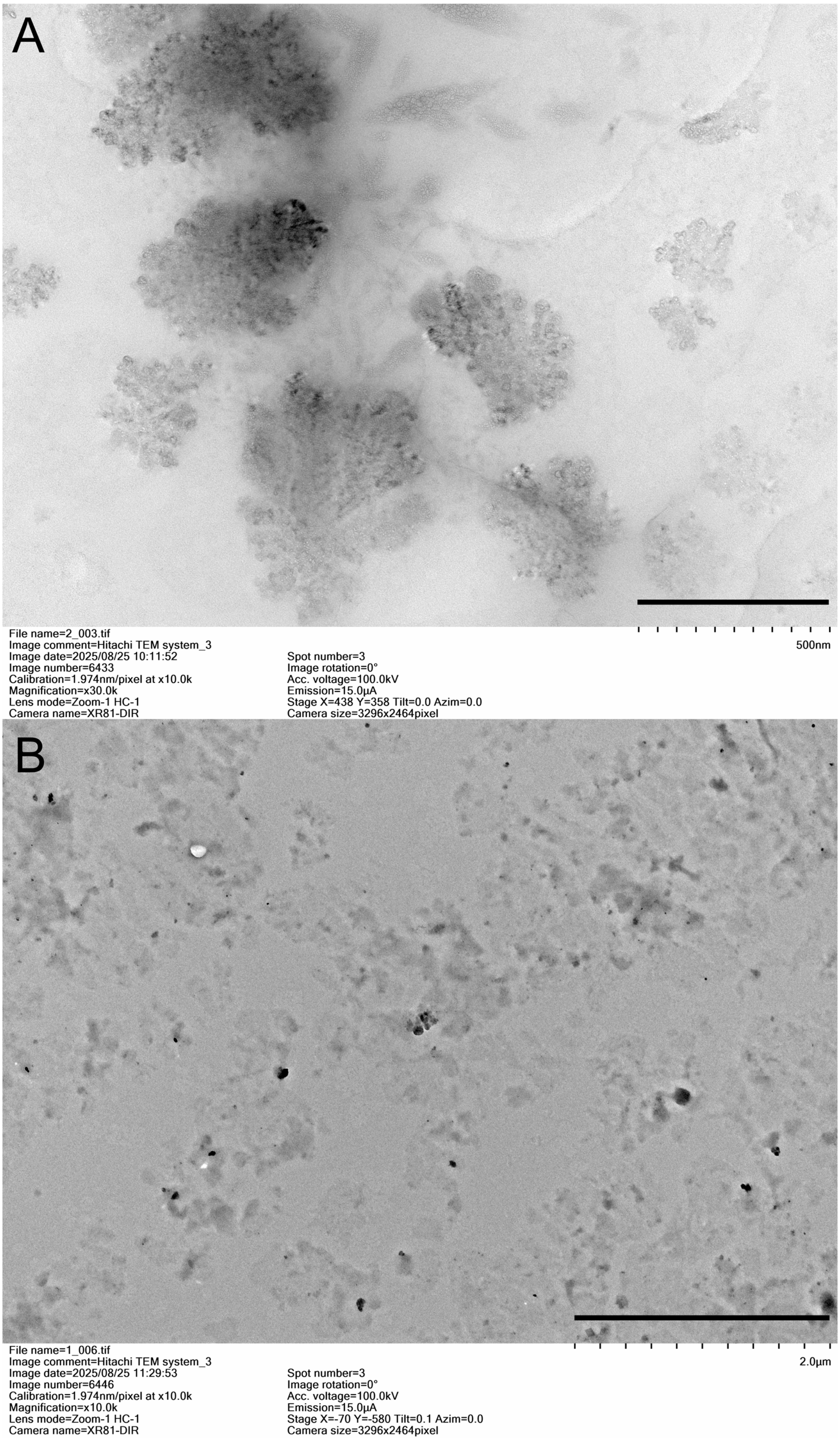
A
500nm
File name=2_003.tif
Image comment=Hitachi TEM system_3
Image date=2025/08/25 10:11:52
Image number=6433
Calibration=1.974nm/pixel at x10.0k
Magnification=x30.0k
Lens mode=Zoom-1 HC-1
Camera name=XR81-DIR
Spot number=3
Image rotation=0°
Acc. voltage=100.0kV
Emission=15.0µA
Stage X=438 Y=358 Tilt=0.0 Azim=0.0
Camera size=3296x2464pixel
B
2.0µm
File name=1_006.tif
Image comment=Hitachi TEM system_3
Image date=2025/08/25 11:29:53
Image number=6446
Calibration=1.974nm/pixel at x10.0k
Magnification=x10.0k
Lens mode=Zoom-1 HC-1
Camera name=XR81-DIR
Spot number=3
Image rotation=0°
Acc. voltage=100.0kV
Emission=15.0µA
Stage X=-70 Y=-580 Tilt=0.1 Azim=0.0
Camera size=3296x2464pixel

Figure S3. TEM images of (A) Eu@BSA nanoparticles and (B) Eu@BSA/Str nanoparticles. Scale bars: A – 500 nm, B – 2000 nm.

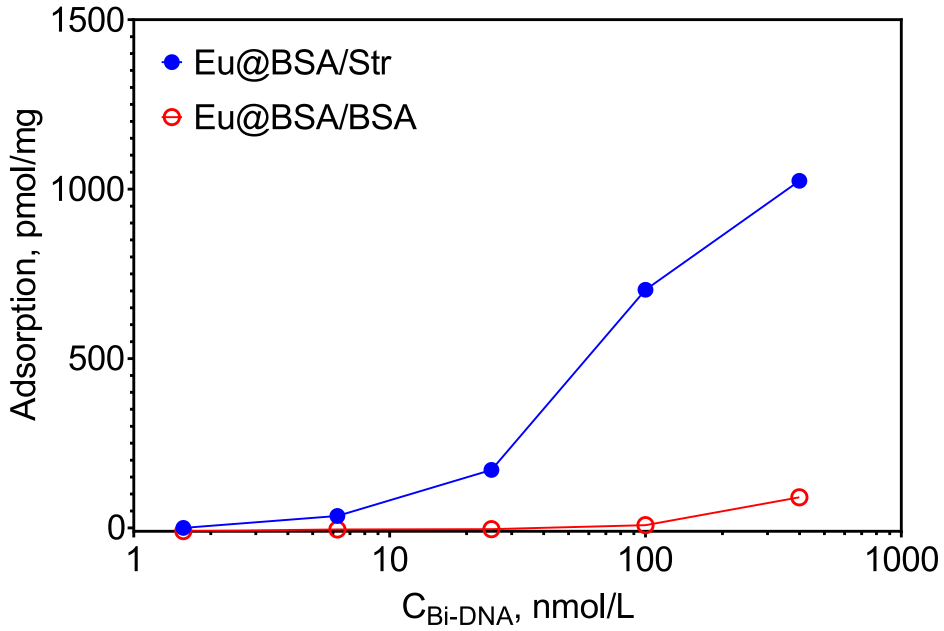

Figure S4. Adsorption of Bi-DNA-FAM on Eu@BSA/Str and Eu@BSA/BSA. Concentration of nanoparticles was 0.1 mg/mL. Reaction medium: 10 mmol/L TRIS-HCl buffer, containing 0.15 mol/L of NaCl, 1% BSA, 0.1 % Tween-20, pH 7.4. Reaction time – 60 min. Temperature – +28 °C.

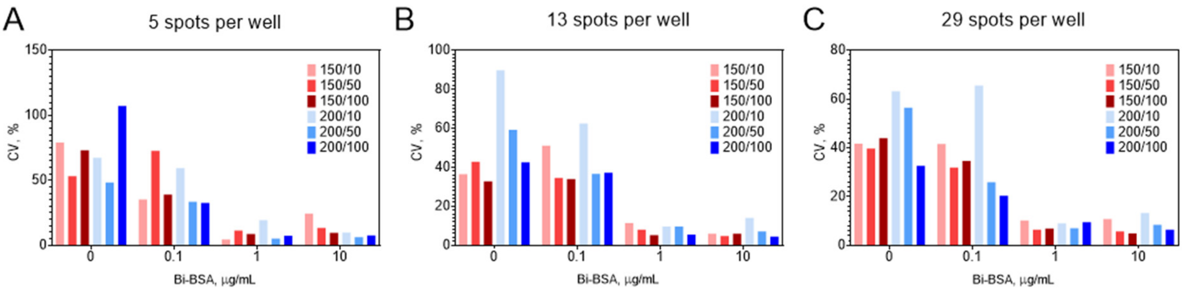

Figure S5. Coefficient of variation of luminescence intensity under different measurement conditions (**A-C**): number of spots per well (indicated above each graph), detector gain, and number of measurements per spot. The legend denotes gain (first value) and number of measurements per spot (second value).

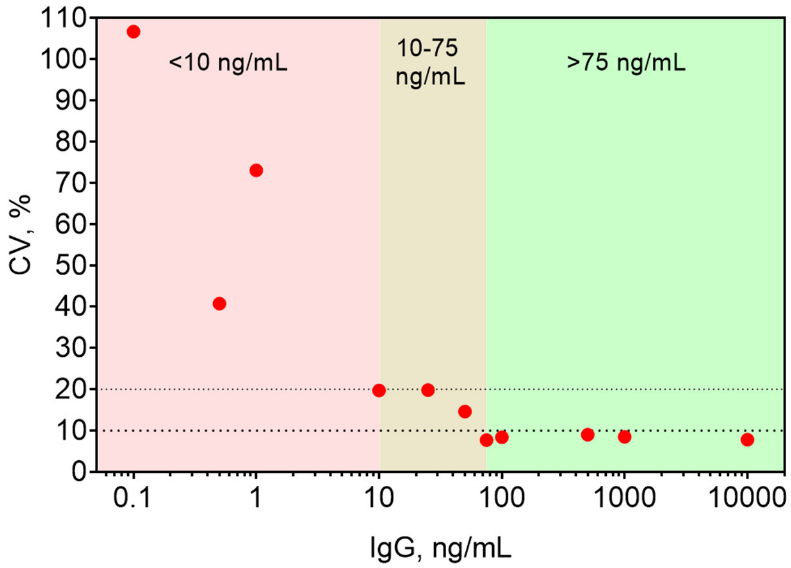

Figure S6. Dependence of luminescence signal coefficient of variation on analyte concentration under optimized measurement conditions: five spots per well, 50 measurements per spot. n = 3; mean ± SD.

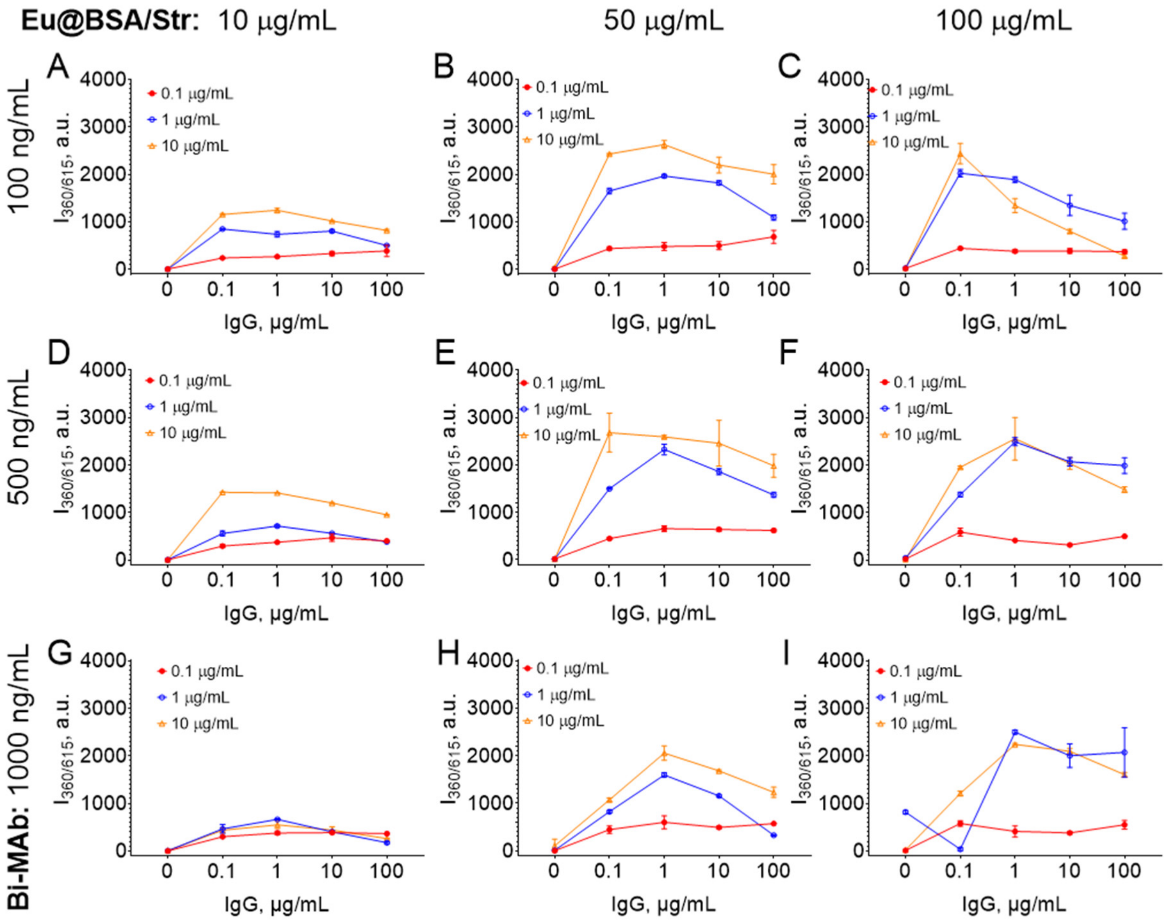

Figure S7. Optimization of capture antibody, detection antibody, and Eu@BSA/Str nanoparticle concentrations for TRLI performance. Legends indicate capture polyclonal antibody concentrations; nanoparticle concentrations are shown above columns for each panel set; biotinylated anti-human monoclonal antibody concentrations are indicated to the left of rows for each panel set. n = 2; mean ± SD.

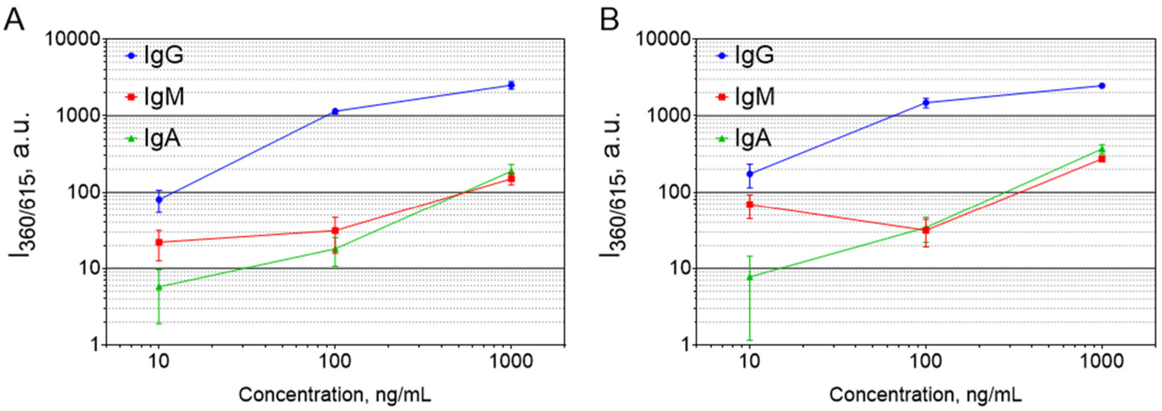

Figure S8. Dose-response curves for IgG, IgM, and IgA in TRLI using biotinylated monoclonal (**A**) and polyclonal (**B**) antibodies. Data represent mean ± SD, n = 4.

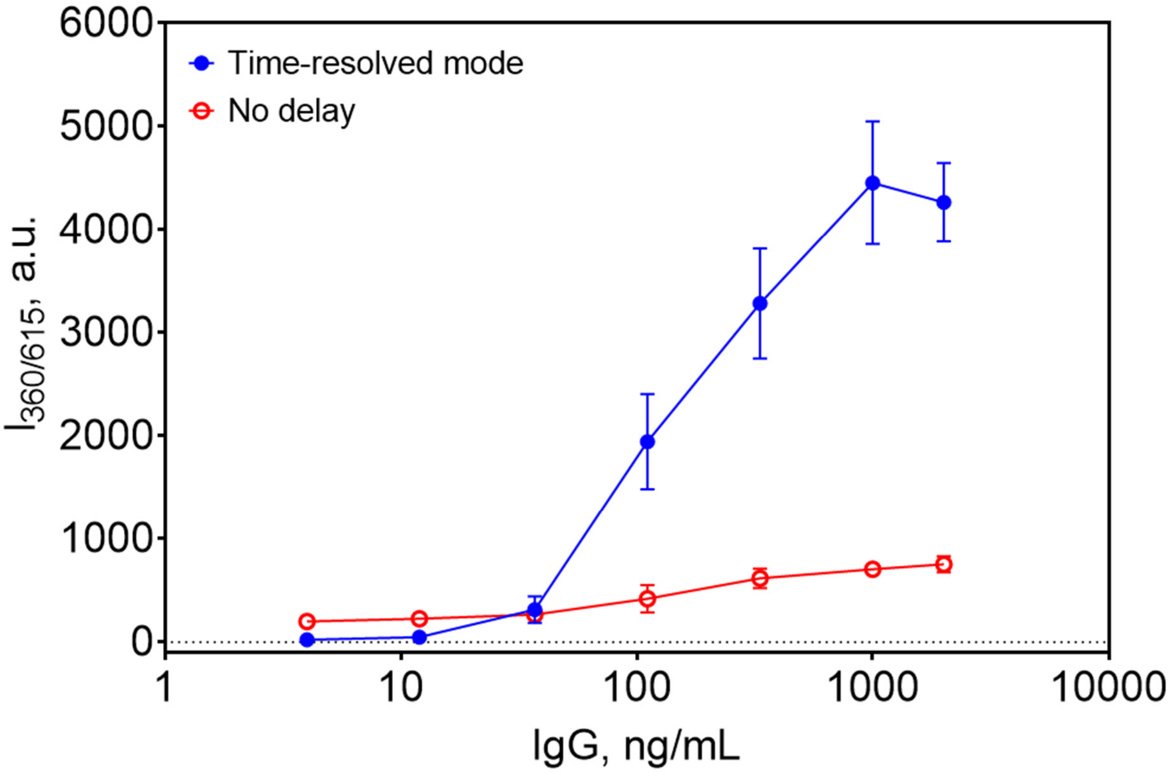

Figure S9. Comparison of IgG standard curves acquired by measuring luminescence signal with and without a time delay (100 μs). In time-resolved mode, mean blank signal was 0.67 a.u.; in non-delayed mode, it was 176.3 a.u. n = 6; mean ± SD.

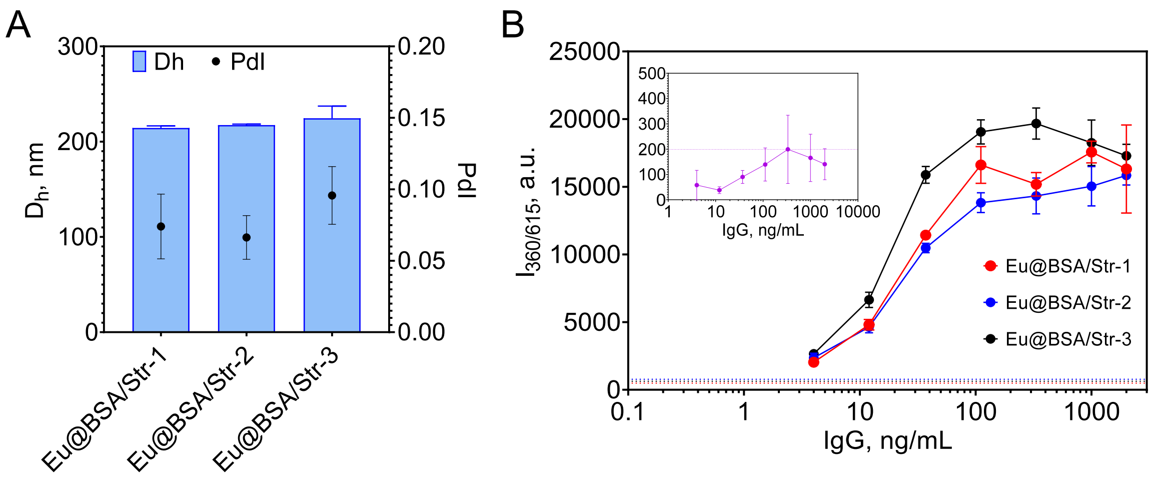

Figure S10. Reproducibility of Eu@BSA/Str properties. A – hydrodynamic diameter and polydispersity index of three batches, n = 3; mean ± SD. B – TRLI with three batches of Eu@BSA/Str. Dotted lines indicate mean value of luminescence intensity of blank sample plus three standard deviations (497.6 for Eu@BSA/Str-1, 772.4 for Eu@BSA/Str-2, and 628.2 for Eu@BSA/Str-3), n = 5; mean ± SD. Inset: TRLI with Eu@BSA/BSA, n = 4; mean ± SD.

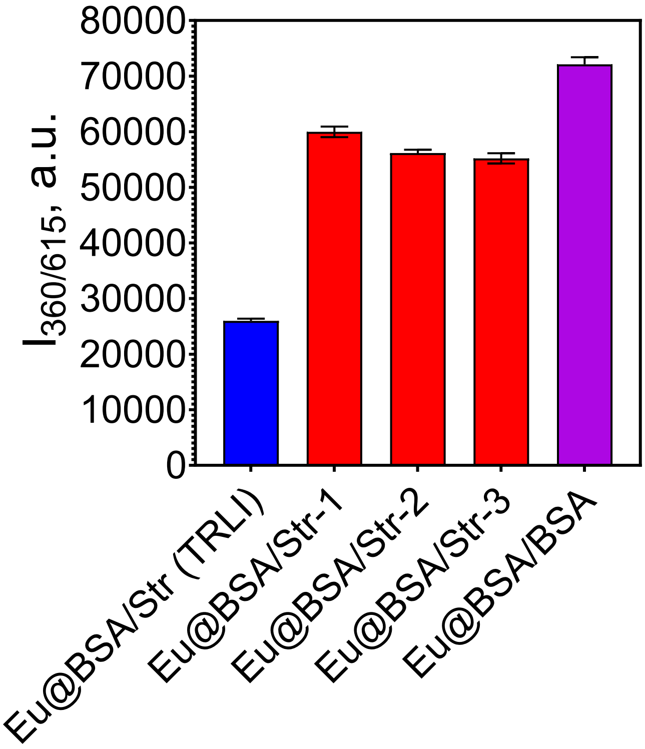

Figure S11. Reproducibility of Eu@BSA/Str luminescence intensity. Eu@BSA/Str (TRLI) – is a nanoparticle sample used in TRLI throuthout the article. Measurement conditions: excitation – 360 nm, emission – 615 nm, delay – 100 μs, acquisition time – 300 μs, gain – 200 a.u. Concentration of nanoparticles: 10 μg/mL, 100 μL per well). n = 3; mean ± SD.

Table S1. Concentrations of IgG standards as determined by different analytical techniques.

	ELISA-1, mg/mL	ELISA-2, mg/mL	Spectrophotometry, mg/mL
IgG "Sigma"	5.124	11.01	8.5
IgG "Mikrogen"	78.8	119.6	107.1

Table S2. Effect of IgM and IgA at concentrations typical of human serum on luminescence intensity in TRLI with biotinylated monoclonal antibodies.

IgG, ng/mL	$I_{360/615, a.u.}$	IgM, ng/mL[1]	$I_{360/615, a.u.}$ added by IgM	Percentage of $I_{360/615, a.u.}$ provided by IgG, %	IgA, ng/mL	$I_{360/615, a.u.}$ added by IgA	Percentage of $I_{360/615, a.u.}$ provided by IgG, %
**10**	80	**1.31**	19.69	24.61	**2.34**	2.16	2.70
**100**	1150	**13.14**	21.23	1.85	**23.42**	6.09	0.53
**1000**	2507	**131.41**	36.65	1.46	**234.19**	45.42	1.81

[1] Expected concentrations of IgM and IgA were calculated based on typical IgG:IgM and IgG:IgA ratios in normal serum. Specifically, the average serum concentration of IgM is 7.61-fold lower than IgG, and IgA is 4.27-fold lower than IgG.

Table S3. Effect of IgM and IgA at concentrations typical of human serum on luminescence intensity in TRLI with biotinylated polyclonal antibodies.

IgG, ng/mL	$I_{360/615,\ a.u.}$	IgM, ng/mL[1]	$I_{360/615,\ a.u.}$ added by IgM	Percentage of $I_{360/615,\ a.u.}$ provided by IgG, %	IgA, ng/mL	$I_{360/615,\ a.u.}$ added by IgA	Percentage of $I_{360/615,\ a.u.}$ provided by IgG, %
**10**	174	**1.31**	39.17	22.51	**2.34**	1.75	1.01
**100**	1485	**13.14**	41.91	2.82	**23.42**	9.52	0.64
**1000**	2472	**131.41**	69.40	2.81	**234.19**	87.19	3.53

[1] Expected concentrations of IgM and IgA were calculated based on typical IgG:IgM and IgG:IgA ratios in normal serum. Specifically, the average serum concentration of IgM is 7.61-fold lower than IgG, and IgA is 4.27-fold lower than IgG.

Table S4. Comparison of human IgG concentrations (mg/mL) in serum samples measured by ELISA and by the luminescent immunoassay.

Sample number	ELISA-1	ELISA-2	TRLI
1	7.23	9.88	9.56
2	9.82	10.03	9.08
3	5.05	8.95	8.18
4	7.10	10.64	6.05
5	4.72	8.97	6.48
6	7.17	12.17	8.60
7	7.94	10.89	10.23
8	10.34	17.30	11.67
9	8.37	9.65	8.62
10	8.10	11.47	11.35
11	7.54	9.11	13.74
12	8.77	10.52	9.38
13	15.80	11.53	10.37
14	6.46	10.42	6.61

15	9.80	11.28	15.81
16	9.18	13.77	17.09
17	7.33	8.83	8.18
18	8.95	9.85	6.88
19	12.38	10.21	10.78
20	4.19	5.12	5.00
21	8.24	6.12	8.50
22	9.53	8.98	8.01
23	10.03	9.05	20.63
24	4.59	6.97	7.67
